# Supplementary material for: Chromothripsis during telomere crisis is independent of NHEJ, and consistent with a replicative origin
Source: Genome Res. 2019 May;29(5):737–49. doi: 10.1101/gr.240705.118 (PMC6499312; doi:10.1101/gr.240705.118)
Supplement: Supplemental Material [file supp_gr.240705.118_Supplemental_file_1.zip › contigs/annotated_contigs/DB46/contig.3.DB46_length_879_mean_cov_9.54721274175.docx]

**DB46_length_879_mean_cov_9.54721274175**

```
AAGGAGAATCAGCAGCTCTACCGCAAGAGTCAGGCAACGCTGGTGCTAGTCAAGGGACTTGGAGAAGGTGACTTATTTTGCCTTTCATC
 >chr11:91069753-91069935 + E=1e-77 p=3e-03
CACCACTACAGACACAGCTGGGGCTTCTCTCACAGGAAGCAGATAGATAGATAAGCATATATATAAGCATATATAGATATAAATATATA

TA|AGCA|GATGAAAAGAGGTGGCTGTCTGATCTGAATAGCTGGAATACTGGGTCAGGAGTGTTTCTGGGAAGTGGATAGGTTTCCTGC
   >chr11:91070156-91070371 + E=2e-117
TGACCTGGCAAGGGAACTGAGGTGGCTCCTACTCTCCCCTCTGTTAAGACTTCAGTGCATTTCACTGACAGCTTCTCTAACCACCTCTG

TCAAAGCTGGGACTTCTGCCCATCATTGGATATTGCAT|TCA|GTGCTCAAAGACAGGGCTTTTTAATTAACCCAATCAGATAAAAATA
                                       >chr11:91071016-91071503 + E=5e-281
AAGCAAAATGAATCAAAAGAAATGAACAAAGTCTCCAAGAATCTGTAAGATGGCCAACCCTAAGAATAATCGGTATTCCTGAGGGAGAC

GAGAAAGCAAAATGTTTGGAAAATTTATTTGAGGAAATGATCGAGATAAACTTCCCTGGCCTTGCTAGAGATTTAGATATCCAAATACA

AGAAGTGCACAGAATTCCTGGGAGATTAGTTGCAAAAAAGTCATCACCAAGGTGTATAGTTATCAAGCTATGTAAAGTCAATATAAAGG

AAAAATTCTAATAGCAATTAGACAAAAGCATCAGATAACTCATAAAGGAAAACCTATCAGACTAACAGCAGTCTTCTCAGCACAAACCG

TAGAAGCCAGAAGAAATTGGGGTATTATCCTTAGCCTCCTTCATACAGAATAACTGTCAGTGAATAACTTTGTATCCAGAAA
```
